# Maternal Arsenic Exposure and Gestational Diabetes: A Systematic Review and Meta-Analysis

**DOI:** 10.3390/nu12103094

**Published:** 2020-10-11

**Authors:** Noemi Salmeri, Roberta Villanacci, Jessica Ottolina, Ludovica Bartiromo, Paolo Cavoretto, Carolina Dolci, Rosalba Lembo, Matteo Schimberni, Luca Valsecchi, Paola Viganò, Massimo Candiani

**Affiliations:** 1 Gynecology/Obstetrics Unit, IRCCS San Raffaele Scientific Institute, 20132 Milan, Italy; salmeri.noemi@hsr.it (N.S.); villanacci.roberta@hsr.it (R.V.); ottolina.jessica@hsr.it (J.O.); bartiromo.ludovica@hsr.it (L.B.); cavoretto.paolo@hsr.it (P.C.); dolci.carolina@hsr.it (C.D.); schimberni.matteo@hsr.it (M.S.); valsecchi.luca@hsr.it (L.V.); candiani.massimo@hsr.it (M.C.); 2 Department of Anesthesia and Intensive Care, IRCCS San Raffaele Scientific Institute, 20132 Milan, Italy; lembo.rosalba@hsr.it; 3 Reproductive Sciences Laboratory, Gynecology/Obstetrics Unit, IRCCS San Raffaele Scientific Institute, 20132 Milan, Italy

**Keywords:** arsenic, arsenic exposure, arsenic toxicity, gestational diabetes mellitus, pregnancy

## Abstract

Gestational diabetes mellitus (GDM) is a metabolic complication associated with adverse outcomes for mother and fetus. Arsenic (As) exposure has been suggested as a possible risk factor for its development. The aim of this meta-analysis was to provide a comprehensive overview of published evidence on the association between As and GDM. The systematic search from PubMed, MEDLINE, and Scopus was limited to full-length manuscripts published in peer-reviewed journals up to April 2020, identifying fifty articles. Ten studies met the inclusion criteria, nine for quantitative synthesis with a total of *n* = 1984 GDM cases. The overall pooled risk was 1.56 (95% Confidence Interval - CI = 1.23, 1.99) with moderate heterogeneity (χ^2^ = 21.95; I^2^% = 64). Several differences among the included studies that may account for heterogeneity were investigated. Stratification for exposure indicator confirmed a positive association for studies assessing urine As. A slightly higher risk was detected pooling studies based in Asia rather than in North America. Stratification for GDM diagnostic criteria showed higher risks when diagnosis was made according to the Canadian Diabetes Association (CDA-SOGC) or World Health Organization (WHO) criteria, whereas a lower risk was observed when adopting the American Diabetes Association (ADA) criteria. These results provide additional evidence for a possible association between As exposure and GDM, although the data need to be interpreted with caution due to heterogeneity.

## 1. Introduction

Gestational diabetes mellitus (GDM), a common metabolic disease that affects up to 14% of pregnant women worldwide, is a glucose intolerance that develops during pregnancy and usually resolves after delivery [1,2]. This condition exposes both mother and fetus to multiple adverse outcomes including an increased likelihood of pre-eclampsia, early delivery, congenital malformations, intrauterine fetal death, fetal macrosomia, polyhydramnios and neonatal hypoglycemia [3,4,5,6]. Furthermore, both GDM mothers and their offspring have higher risk of developing type 2 diabetes mellitus (DM2) and cardiovascular diseases [7,8,9,10]. Since traditional well-known GDM risk factors such as maternal age, obesity, lifestyle and ethnicity [11,12,13] do not clearly explain the prevalence of the disease in pregnancy, there has been a growing interest in the hypothesis that some environmental factors may be implicated in GDM pathogenesis. Among all the widespread naturally occurring pollutants, Arsenic (As) is one of the potential candidates [14,15,16]. Millions of people are chronically exposed to As, primarily through contaminated drinking water at concentrations above the World Health Organization (WHO) guideline limit of 10 μg/L [17,18] or by ingestion of some foods such as rice or seaweed. Inorganic As, largely consisting of arsenate and to a lesser extent arsenite [19], is either metabolized and methylated in the liver to both monomethylarsonic acid (MMA) and dimethylarsinic acid (DMA) or excreted unchanged in urine [20]. This metal seems to interfere with different processes including oxidative stress, signal transduction and gene expression, resulting in the growth hormone/insulin-like growth factor axis disruption and pancreatic beta-cell dysfunction [21,22,23,24].

Several studies have found an association between GDM and As levels in maternal blood, urine and meconium, supporting the possibility that a high level of As exposure might predispose to maternal GDM. However, the data obtained so far are quite inconsistent [14,25,26,27,28,29,30,31,32,33].

To offer an overview of the evidence available in the literature, we conducted a systematic review and meta-analysis on the plausible link between maternal As exposure and the risk of developing GDM.

## 2. Materials and Methods

This systematic review and meta-analysis were performed according to the Preferred Reporting Item for Systematic Reviews and Meta-analysis (PRISMA) guidelines [34]. The study protocol was registered and accepted in PROSPERO before starting the data extraction (ID CRD42020195667). No Institutional Review Board approval was needed.

### 2.1. Search Strategy and Study Selection

We performed an advanced, systematic search of the online medical databases PubMed, Medline and Scopus using the following keywords: “arsenic” and “arsenic exposure” in combination with “gestational diabetes mellitus” or “diabetes in pregnancy”. Specific tools available in each database such as MeSH terms were used to optimize search output. Only manuscripts written in English and published in peer-reviewed journals up to April 2020 were included and duplicates were removed by using Endnote software version X9 (Clarivate Analytics, Philadelphia, USA, 2013). The potential eligibility of articles was first assessed by screening titles and abstracts. Then, full-text manuscripts were obtained and the final decision for inclusion was made after detailed examination of the articles. In order to identify any additional relevant citations, we also checked the reference lists of the eligible articles. The electronic search, the study selection and the eligibility for qualitative synthesis were independently assessed by two authors (R.V. and C.D). An independent author (N.S) assessed the eligibility for quantitative synthesis. Disagreements were resolved by discussion with a fourth reviewer (J.O.).

### 2.2. Inclusion Criteria

The following predefined inclusion criteria were used to screen citations for eligibility: (i) exposure to As was assessed through an appropriate exposure indicator (serum/plasma As, urinary As, toenail As, tap water As, meconium As); (ii) risk estimates were provided using odds ratio (OR) or relative risk (RR) with the corresponding 95% confidence interval (CI); (iii) study design limited to analytical studies (cross-sectional, case-control, cohort, ecologic or correlational); (iv) outcome of interest was GDM and diagnosis of GDM was confirmed by a positive glucose challenge test (GCT, 50 gr) and/or a positive oral glucose tolerance test (OGTT, 75/100 gr), according to the diagnostic criteria recommended by either the American Diabetes Association (ADA), the World Health Organization (WHO), the French National College of Obstetricians and Gynecologists (Collège National des Gynécologues et Obstétriciens Français, CNGOF), or the Canadian Diabetes Association and the Society of Obstetricians and Gynecologist of Canada (CDA-SOGC) [35,36,37,38,39]. 

We excluded descriptive studies (case-report and case-series) and studies not reporting original results (reviews, abstracts, editorials, comments) as well as those dealing with the pathological condition of altered blood glucose levels not satisfying the diagnostic criteria for GDM (i.e., impaired fasting glucose (IGT)). Finally, studies were excluded from the quantitative synthesis (meta-analysis) if a comparable estimation of effect size was not provided or in the sensitivity analyses.

### 2.3. Data Extraction

The following data from studies included in the quantitative synthesis were collected and tabulated by three independent reviewers (N.S., C.D. and R.V.) using a standardized data extraction form: (i) first author name, (ii) publication year, (iii) study country, (iv) study period, (v) study design, (vi) sample size, (vii) age and demographic data of the sample, (viii) number of cases, (ix) diagnostic method used to define cases, (x) exposure, (xi) exposure indicator (serum/plasma As, urinary As, toenails As, tap water As, meconium As), (xii) time of pregnancy when exposure was detected, (xiii) confounding variables in multivariate analysis, and (xiv) risk estimates with 95% CI.

### 2.4. Assessment of Risk of Bias

Two review authors (R.V. and C.D.) independently assessed the risk of bias by using the risk of bias tool for cohort studies developed by the Clarity Group (Supplementary Figure S1) [40].

We classified the possible sources of bias as definitely yes (low risk of bias), probably yes (moderate risk of bias), probably no (serious risk of bias), and definitely no (critical, high risk of bias), and then we assessed a comprehensive risk of bias judgment for each study included in our review.

In the case of disagreements, resolution was achieved by discussion with a third reviewer (J.O.).

### 2.5. Data Analysis

Risk estimates with 95% CI were extracted by an independent reviewer (N.S.) from the original works. Almost all the studies included in the quantitative analysis presented odds ratios (ORs) and their 95% CIs. Relative risks (RRs) were converted in ORs [41]. In studies reporting results for several confounding parameters, we used the data adjusted for the largest number of factors. In studies reporting risk estimates for tertiles/quartiles of exposure, we considered the data for the highest.

Multivariate-adjusted risk estimates were transformed into log ORs and were pooled together using the generic inverse-variance approach as the model estimator with both fixed and random effect analysis. To incorporate the estimate of the pooled effect measure in the between-study variance (τ^2^), the random-effect model suggested by DerSimonian and Laird was preferred for the quantitative synthesis of all included studies [42]. A *p*-value < 0.05 was interpreted as statistically significant. Sensitivity analyses were conducted by omitting one study at a time to explore the weight of each work in estimating pooled risks. 

Statistical heterogeneity of the intervention effects was assessed with χ^2^ test and I^2^ statistics. I^2^ index values were interpreted as follows: insignificant heterogeneity if I^2^ was 0–25%, low heterogeneity for I^2^ 25–50%, moderate heterogeneity when I^2^ 50–75% and high heterogeneity, whereas I^2^ was greater than 75% [43]. A low *p*-value (<0.10) from the χ^2^ test indicated heterogeneity [44]. 

Potential publication bias was investigated by plotting the natural logarithm of the estimated OR (lnOR) against its standard error (SE). Asymmetry of the funnel plot was verified using the linear regression method proposed by Egger et al. [45].

Subgroup analyses were performed following the guidelines suggested by Wang et al. [46]. Risk estimates were combined using both fixed and random effect models. An a priori-defined subgroup analysis based on study design (cross-sectional, case-control, cohort, correlational) was performed. Subgroup-analysis based on the exposure assessment method (serum/plasma As, urinary As, toenail As, tap water As, newborn meconium As), study country (North America, South America, Asia, Europe), and diagnostic criteria for GDM (ADA, WHO, CNGOF, CDA-SOGC) were then performed to investigate the possible causes of statistical and clinical heterogeneity. All subgroup analyses were implemented when at least two studies could be included.

Statistical analysis was performed using RevMan software version 5.3 (Copenhagen: The Nordic Cochrane Center, The Cochrane Collaboration, 2014).

## 3. Results

### 3.1. Literature Search

The literature search identified 50 articles: among them, 10 met the inclusion criteria and the following characteristics were extracted [14,25,26,27,28,29,30,31,32,33]. The main characteristics of the included studies are summarized in Table 1. All the included studies were published recently, between 2015 and 2020. More than a half (six out of ten) were cohort studies, two were cross-sectional studies, one was a retrospective case-control study nested in a cohort and one was a correlational study.

**Figure 1:**
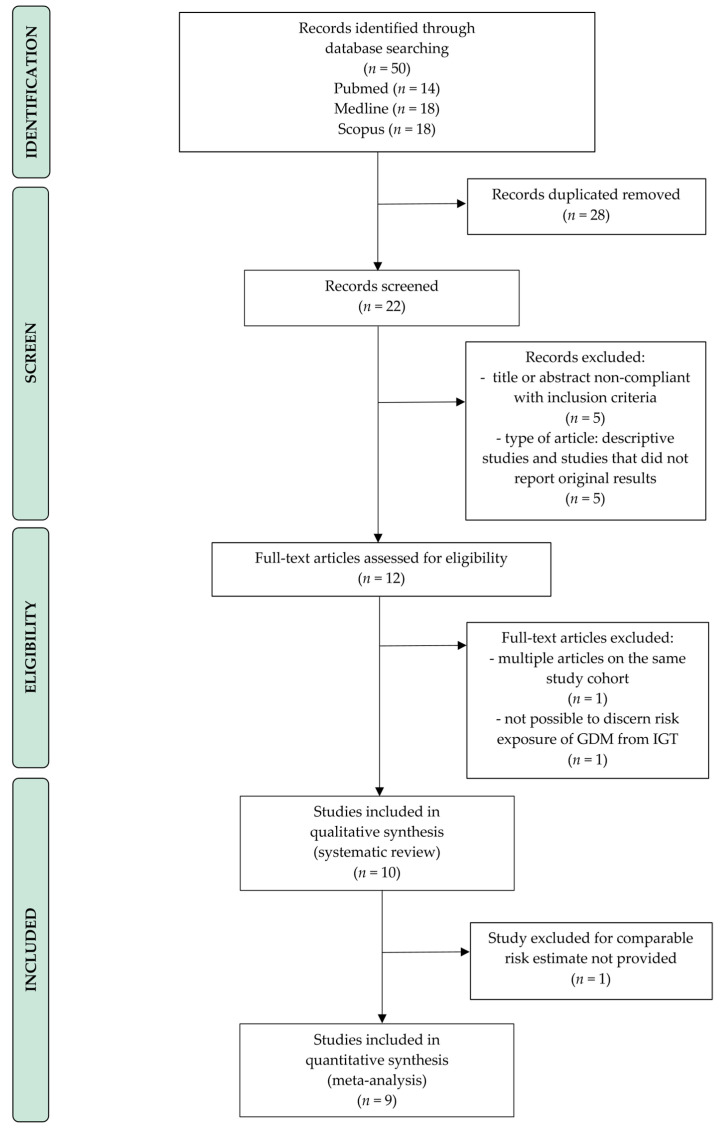
Flow diagram of the search strategy, screening, eligibility and inclusion criteria. Abbreviations: GDM, Gestational diabetes mellitus; IGT, impaired glucose tolerance.

The flowchart of the systematic review is available in Figure 1 (PRISMA template). The risk of bias of the included studies are summarized in Supplementary Figure S1.

### 3.2. Description of Studies

The plausible association between GDM and As exposure was assessed by the analysis of different human samples. Three studies collected blood samples, five papers analyzed urine samples, two evaluated arsenic concentration in home tap water, only one study measured As concentration in the meconium, and one paper in urine samples, home tap water, and toenails. 

#### 3.2.1. Arsenic in Blood Samples

Shapiro et al. used As in first trimester blood samples as an indicator of exposure, finding elevated odds of GDM in the highest quartile of As exposure in the adjusted analysis (adjusted odds ratio (aOR) = 3.7; 95% CI = 1.4, 9.6) [14]. 

Similar results were obtained by Xia et al., who evaluated As levels in blood samples in the first and second trimester and cord blood, finding an association between GDM and As levels only for the fourth quartile of the first trimester samples (aOR = 1.71; 95% CI = 1.23, 2.38). Stratified analyses showed the association was largely limited to normal maternal age (aOR = 1.90; 95% CI = 1.19, 3.04) and normal weight women (aOR = 1.77; 95% CI = 1.18, 2.66) [25]. 

The cohort study conducted by Wang et al., which evaluated blood samples taken the day after delivery, showed an increased risk of GDM for the second tertile (aOR = 1.49; 95% CI = 1.11, 2.01). This risk was even higher among women with low pre-pregnancy BMI (<18.5 kg/m^2^) (aOR = 2.69; 95% CI = 1.04, 6.95) and high pre-pregnancy BMI (≥24 kg/m^2^) (aOR = 2.68; 95% CI = 1.36, 5.27) in the second tertile [26]. 

#### 3.2.2. Arsenic in Urine Samples

The prospective cohort study by Wang et al., which evaluated the exposure to multiple metals in pregnancy, showed a significant and positive association between creatinine-adjusted urinary arsenic.

Levels and GDM (*p* = 0.026). However, a significant association between arsenic concentration and risk of GDM was found only in the single metal model (*p* = 0.019) without any validation in the multiple-metals model analysis (including urinary nickel, antimony, cadmium, cobalt, vanadium) [31].

Ashley-Martin et al. analyzed urinary metabolites (DMA and arsenobetaine) of As, stratifying results for urinary specific gravidity. They found a significantly increased risk of GDM (aOR = 3.86; 95% CI = 1.18, 12.57) in women with DMA concentration higher than 3.52 μg As/L (third tertile). Interestingly, the aOR was even higher when the analysis was restricted to women carrying male infants (aOR = 4.71; 95% CI = 1.05, 21.10) [28].

The study conducted by Khan et al. demonstrated that As level in urine might predict the likelihood of having GDM [30]. However, both Farzan et al. and Munoz and colleagues did not draw similar conclusions, finding no association between urinary As concentrations and GDM [27,29]. 

#### 3.2.3. Arsenic in Tap Water Samples

The findings from Farzan et al. found a close relationship between As exposure via home well water and risk of GDM: each 5 μg/L increase in As concentration in home well water was associated with a 10% increased odd of GDM (aOR = 1.1; 95% CI = 1.0, 1.2). This association was largely limited to obese women (BMI ≥ 30 kg/m^2^) (aOR = 1.7; 95% CI = 1.0, 2.8) [27]. 

The French correlational study carried out by Marie and colleagues [33] provided additional evidence on the association between As concentration in tap water samples and incidence of GDM. Women exposed to As level ≥10 μg/L (As + group) had a higher risk of developing GDM than those exposed to As level ≤10 μg/L (As – group) (aOR = 1.62; 95% CI = 1.01, 2.53). Stratified analysis of pre-pregnancy BMI showed a positive association only for obese or overweight women (BMI ≥ 25 kg/m^2^) (aOR = 2.30; 95% CI = 1.13, 4.50). 

#### 3.2.4. Arsenic in Meconium Samples

Only one study investigated the link between GDM and As exposure in meconium, finding a higher concentration of the metal in studied cases when compared to controls. Arsenic levels were positively associated with maternal GDM with aORs of 3.28 (95% CI = 1.24, 8.71), 3.35 (95% CI = 1.28, 8.75) and 5.25 (95% CI = 1.99, 13.86) for the second, third, and fourth quartiles, respectively [32]. 

#### 3.2.5. Arsenic in Toenail Samples

One of the included studies investigated the association between As exposure and the risk of GDM measuring As concentrations in toenails. A positive and statistically significant association was observed: each 100% increase in toenail As was associated with a nearly four-fold increased risk of GDM (aOR = 4.5), despite the wide confidence interval (95% CI = 1.2, 16.6) [27]. 

### 3.3. Meta-Analysis

**Figure 2:**
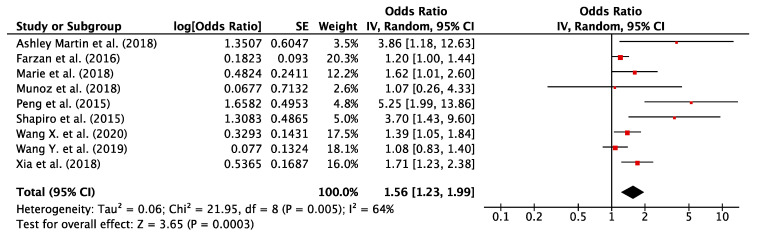
Forest plot of all studies included in the quantitative-synthesis (*n* = 9). The point estimate for each study is represented by a red square where the size of the square is proportional to the weight of the study in the meta-analysis and the 95% CI is symbolized by an horizontal line. The total effect with 95% CI is represented by a black diamond. The results of the pooled analysis demonstrate that As exposure increased the risk of developing GDM (OR = 1.59; 95% CI = 1.23, 1.99). Abbreviations: CI, confidence interval; df, degrees of freedom; IV, inverse variance; SE, standard error.

**Figure 3:**
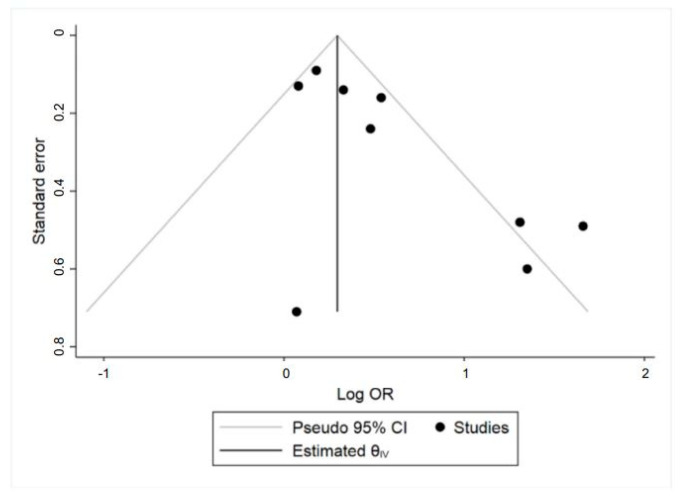
Funnel plot of all studies included in the quantitative-synthesis (*n* = 9). Visual inspection demonstrates slightly high publication bias, as confirmed by Egger’s test (t =3.00; *p* = 0.02). Abbreviations: CI, confidence interval; OR, odds ratio.

The forest plot of the meta-analysis including all studies for As exposure and the risk of GDM is reported in Figure 2. Funnel plot for publication bias is illustrated in Figure 3. The study conducted by Khan et al. was excluded from the quantitative synthesis as it was not possible to obtain a comparable estimation of effect size [30].

For all the included studies (*n* = 9) the pooled OR calculated according to the random effect model was 1.56 (95% CI = 1.23, 1.99), with obvious moderate heterogeneity (χ^2^ = 21.95; *p* = 0.005; I^2^% = 64) and slightly high publication bias (Egger’s test: t = 3.00; *p* = 0.02) [14,25,26,27,28,29,31,32,33]. The positive association of maternal As exposure with GDM yielded a statistically significant result (*p* for effect = 0.0003). The meta-analysis performed using the fixed effect model showed quite similar results (OR = 1.34; 95% CI = 1.20, 1.51; *p* for effect <0.00001). Sensitivity analysis conducted by omitting one study at time (*n* = 8) revealed that the result of the pooled analysis was quite robust. 

#### Subgroups Analyses

The results of the different meta-analyses performed are reported in Table 2. An a priori-defined subgroup analysis based on study design showed less inconsistency/ heterogeneity (χ^2^ = 1.86; *p* = 0.17; I^2^% = 46) and high pooled risk (OR = 2.28, 95% CI = 0.92, 5.64) when combining data from cross-sectional studies rather than when pooling data from the cohort studies (heterogeneity: χ^2^ = 13.73; *p* = 0.008; I^2^% = 71; effect estimate: OR = 1.16; 95% CI = 1.07, 1.26). 

Further analyses were performed to investigate the possible causes of heterogeneity, stratifying studies according to exposure indicator, study country, and diagnostic criteria for GDM.

We found low heterogeneity when combining studies assessing urine As (χ^2^ = 4.20; *p* = 0.24; I^2^% = 29), moderate heterogeneity when pooling studies measuring tap water As (χ^2^ = 2.49; *p* = 0.11; I^2^% = 60), and high heterogeneity when studies based on blood As were combined together (χ^2^ = 8.87; *p* = 0.01; I^2^% = 77). The pooled effect estimates according to stratification by exposure indicator carried quite similar results for urine and blood As (urine As: OR = 1.39; 95% CI = 1.07, 1.82; blood As: OR = 1.35; 95% CI = 1.11, 1.65), whereas a minor association was found for tap water As (OR = 1.11; 95% CI = 1.02, 1.21). 

When combining data from different study countries, we found a similar high heterogeneity for studies conducted in North America (χ^2^ = 8.57; *p* = 0.01; I^2^% = 77) and in Asia (χ^2^ = 12.32; *p* = 0.006; I^2^% = 76). The pooled risk estimate was slightly higher for studies based in Asia (OR = 1.37; 95% CI = 1.17, 1.62), rather than in North America (OR = 1.28; 95% CI = 1.07, 1.53). For studies based in North America, a sensitivity analysis was conducted by omitting Shapiro et al., since redundancy of data between Shapiro et al. and Ashley-Martin et al. could not be excluded [14,28]. 

For studies based in Asia, a sensitivity analysis was conducted by omitting Peng et al., in light of the methodological differences in study design and exposure indicator from the other studies included in the analysis [25,26,31,32]. Sensitivity analyses reduced heterogeneity, confirming that results were quite robust.

Stratification by diagnostic criteria of GDM showed higher pooled risk estimates when diagnosis of the disease was made according to CDA-SOGC criteria (OR = 3.76; 95% CI = 1.79, 7.91) or WHO criteria (OR = 3.13; 95% CI = 1.41, 6.95) rather than with ADA criteria (OR = 1.27; 95% CI = 1.12, 1.43). We found no heterogeneity when combining studies where GDM diagnosis was established with CDA-SOGC criteria (χ^2^ = 0.00; *p* = 0.96; I^2^% = 0), low heterogeneity when pooling studies adopting ADA diagnostic criteria (χ^2^ = 5.37; *p* = 0.15; I^2^% = 44), and moderate heterogeneity when studies defining cases according to WHO diagnostic criteria were combined together (χ^2^ = 3.36; *p* = 0.07; I^2^% = 70). For studies where diagnosis of GDM was based on ADA criteria, a sensitivity analysis was performed by omitting Farzan et al. [27] since this was the only study where cases were identified with both the one-step and the two-step approaches of ADA diagnostic criteria [35]. The sensitivity analysis showed that the result was quite robust. 

For all the subgroup analysis performed, visual inspection of funnel plots did not detect substantial asymmetries and yielded little evidence of publication bias (Supplementary Figure S2). However, due to the low number of publications, such bias could not be entirely ruled out. 

## 4. Discussion

The overall results from this meta-analysis provide evidence for an association between exposure to As and GDM, underlining the possible disrupting role of As in glucose metabolism. However, the few number of studies available and the strong heterogeneity existing among them suggests caution in the interpretation of the data.

Gestational diabetes mellitus is a common complication of pregnancy characterized by a dysfunction of pancreatic β-cells on a background of chronic insulin resistance [47]. In normal pregnancy, insulin sensitivity physiologically changes depending on gestational age; in early gestation, the sensitivity increases, promoting glucose uptake in adipocytes in order to store energy for later pregnancy [48]. In the second half of pregnancy, the insulin sensitivity decreases, improving circulating glucose levels for fetal growth requests [49]. In the case of GDM, the β-cells became dysfunctional, losing the ability to adequately control glucose blood concentration. According to the most recent International Diabetes Federation (IDF) estimates, GDM affects approximately one out of seven pregnancies [2]. Since traditional risk factors do not clearly explain the worldwide increasing incidence of the disease, there is a growing interest in the exposure to untraditional risk factors such as environmental contaminants. Among them, the interference with critical steps in glucose metabolism induced by As metabolites has been quite extensively investigated [50]. 

Arsenic environmental pervasiveness makes its exposure a daily event [51]. As it is comprised of numerous inorganic and organic species, each of them induces a heterogeneous degree and type of toxicity [52]. Arsenate and arsenite are the two most common forms of inorganic As found in drinking water, rice, and seaweed. The components of organic As (mainly found in seafood) such as arsenosugars, arsenolipids, and arsenobetaine (AsB) have historically been thought to be relatively nontoxic and excreted largely unchanged in urine [19,52]. The inorganic As compound has multiple properties that may adversely affect glucose homeostasis [50]. Arsenate can substitute phosphates in the synthesis of adenosine triphosphate (ATP), altering the ATP-dependent insulin secretion. It can form covalent bonds with the disulfide bridges of insulin, insulin receptors, glucose transporters (GLUTs), and enzymes involved in glucose metabolism (e.g., pyruvate dehydrogenase and α-ketoglutarate dehydrogenase). Moreover, it can alter the expression of peroxisome proliferator-activated receptor γ (PPARγ), a nuclear hormone receptor involved in insulin activation. However, the pancreatic β-cell dysfunction induced by oxidative stress and by interferences in signal transduction or gene expression seems to be the main molecular mechanisms responsible for arsenic-induced diabetes mellitus. As exposure induces the formation of superoxide that, through the interaction with uncoupling protein 2 (UCP2), theoretically impair insulin secretion and create a state of oxidative stress that leads to amyloid deposition in β-cells, causing their progressive destruction [24].

On the basis of these observations, recently, several studies have tested the hypothesis that maternal As exposure may also increase the risk of developing adverse maternal metabolic outcomes such as GDM [14,25,26,27,28,29,31,32,33].

Three studies reported statistical support to the relationship between As exposure and risk of GDM using total As in blood as the exposure variable [14,25,26]. The assessment of total blood As may represent an overestimation of the exposure because of the different toxicity of inorganic and organic As species [15,53]. Moreover, As levels in blood have a short half-life, possibly leading to mistakes in the assessment of exposure [54]. On the other hand, blood As can reach a steady-state status in chronically exposed people, also reflecting long-term exposure levels [55]. The current meta-analysis showed a significant association between blood As level and GDM only for the highest levels of exposure (OR = 1.35; 95% CI = 1.11, 1.65). However, some factors may have influenced these results such as the different study populations, different pregnancy trimesters of sampling, stratification of level of exposure, confounding factors considered, and the inability to rule out the contributions of organic As to total As. In any case, a considerable heterogeneity was detected among the studies (I^2^% = 77). 

Five studies used urine samples in order to assess the association between exposure to As and GDM [27,28,29,30,31]. Urinary As levels reflect the As exposure over the past 2–3 days, representing a short-term measure of both inorganic and organic As species [55]. Three studies out of five showed a significant and positive association between As concentration and GDM [28,30,31]. An increased risk of GDM in women with urinary metabolite DMA concentrations higher than 3.52 μg As/L during the first trimester was found by Ashely-Martin and coworkers [28]. However, it is likely that those results were influenced by several issues including the different timing of urine sampling, different stratification of level of exposure, different confounding factors, and urinary markers of exposure considered. The main finding of this meta-analysis revealed a significant association between urinary As level and GDM (OR = 1.39; 95% CI = 1.07, 1.82) with a low heterogeneity among the included studies (I^2^% = 29), suggesting a possible more accurate assessment of As exposure when using urine As as the exposure indicator. 

The association between exposure to As in tap water and GDM was assessed in two studies, which reported a significant association [27,33]. In line, the current meta-analysis showed a significant moderate association between tap water As level and the disease (OR= 1.11; 95% CI = 1.02, 1.21), although lower than that of the other sources, with a moderate heterogeneity among the included studies (I^2^ % = 60). Water As level represents a valid exposure measure for inorganic As if it is the primary source of exposure and individual water intake levels are known. However, it might underestimate the exposure among people with high inorganic As intake from foods (e.g., rice, poultry, fruits, and dairy product), leading to altered exposure assessment [56,57].

Arsenic levels in maternal toenail samples and fetal meconium were also analyzed by two different studies that found a significant association [27,32]. Toenail As is a valid biomarker of inorganic As exposure since it reflects the exposition of 6–12 months prior to sample collection, providing a more long-term exposure measure compared to urine samples [58]. Furthermore, the use of meconium offers even more interesting advantages such as its production from the 12th week of gestation to childbirth (the longest term exposure indicator), the non-invasive sampling, and its capability to reflect maternal and fetal exposure simultaneously [59].

Grouping of the studies by study country did not reduce heterogeneity either for studies conducted in North America (I^2^% = 77) or when pooling Asian studies together (I^2^% = 76). Moreover, a substantial reduction in heterogeneity among Asian studies (I^2^% = 58) was observed when the analysis was performed by omitting the study by Peng et al. [32] because of its intrinsic methodological differences from the other studies included in the stratification, confirming that the results were quite robust. A significant positive association was detected both in North American and in Asian studies, however, with a slightly higher risk estimate for studies based in Asia rather than in North America (OR = 1.37; 95% CI = 1.17, 1.62 and OR = 1.28; 95% CI = 1.07, 1.53, respectively). These findings could be explained by different ethnic, geographic, and dietary arsenic exposures among countries [60]. Moreover, the frequencies of different genetic polymorphisms of the main enzymes involved in the arsenic metabolism such as purine nucleoside phosphorylase (PNP), arsenic methyltransferase (AS3MT), and glutathione-S-transferases (GSTs) vary worldwide, depending on ethnicity/race [61,62,63]. In any case, the low number of studies included in the stratifications led to not very accurate risk estimates in those analyses. 

Combining studies according to the different criteria adopted for GDM diagnosis, we found no heterogeneity among studies based on CDA-SOGC diagnostic criteria (I^2^% = 0). Nevertheless, both studies included according to this stratification [14,28] extracted study participants from the Maternal-Infant Research on Environmental Chemicals (MIREC) longitudinal birth cohort of Canada, with the consequence that the result of this analysis needs to be interpreted with caution. Indeed, a substantial reduction in heterogeneity was also observed when pooling studies adopting ADA diagnostic criteria (I^2^% = 44), while the main source of heterogeneity came from studies based on WHO diagnostic criteria (I^2^% = 70). This finding could be explained by a different definition of cases in the study based in Chile [29], which actually adopted a modified version of the WHO diagnostic criteria established by the Ministry of Health, Chile [64]. A significant strong association was observed when diagnosis of the disease was made by the CDA-SOGC criteria (OR = 3.76; 95% CI = 1.79, 7.91) or WHO criteria (OR = 3.13; 95% CI = 1.41, 6.95), whereas a lower yet still positive association was observed among studies defining GDM cases according to ADA criteria (OR = 1.27; 95% CI = 1.12, 1.43). These findings could be explained by the marked differences among these criteria in blood glucose assessment tests (GCT 50 g, OGTT 75 g, OGTT 100 g) and thresholds, the period of pregnancy in which the test is recommended, the screening approach (universal or selective), and the screening steps (one or two step) to confirm GDM diagnosis [35,36,37,38,39]. Indeed, an internationally consistent definition of GDM remains elusive despite the attempts at building a consensus [65]. The lack of consistency in screening and diagnosis of GDM within and between countries leads to a substantial difficulty in estimating GDM prevalence worldwide. As a matter of fact, identification of potential environmental risk factors linked to the disease remains challenging [66].

The major strength of the current meta-analysis is that it offers an up-to-date overview for those who approach this topic. Indeed, a significant association between As exposure and diabetes has been already established in the non-pregnant population [24,50]. In recent years, only a few studies investigating the link between As exposure and the risk of GDM have been published. The present study is, to our knowledge, the first comprehensive overview of available evidence on the association between As and GDM.

To properly interpret the results, it needs to be emphasized that a causal relationship between As exposure and GDM could be demonstrated only if the occurrence of As exposure was prior to the development of GDM. As already mentioned, the various As biomarkers have several strengths and limitations and reflect a different time of exposure to As. Therefore, considering that the half-life of As in blood is short (several hours) [54], we included in our meta-analysis the data from one study that collected samples during the first trimester [14], and only the data from the first trimester samples of the study assessing As levels in all trimesters of gestation [25]. The third of the studies included in the analysis [26] collected samples the day before delivery, so after the diagnosis of GDM. Moreover, since the main sources for blood As are drinking water and food and the authors declared a relatively stable consumption of them by women during pregnancy [26], we considered women included in this study as chronically exposed to As. Since the steady-state status reached by those women reflects a long-term exposure [55], it should be plausible to consider the causal relationship between prior As exposure and GDM development. Urinary As is a short-term biomarker (2–3 days) [55], thus we excluded from our meta-analysis one article where no timing of exposure was provided [30]. All the selected articles assessed As exposure by urinary levels in the first [28] or in the early second trimester [27,29,31] (so prior to GDM diagnosis), making a causal relationship possible between prior As exposure and subsequent GDM development. For both articles assessing tap water As, a relationship between earlier As exposure and later GDM diagnosis could be supposed. One study enrolled women at 24–28 gestational weeks, who reported using the same water at their residence since their last menstrual period [27], whereas in the other included study, the period of exposure for each woman was the entire year preceding the date of delivery, thus comprising the periconceptional period and all the trimesters of pregnancy [33]. Both toenail As and meconium As are long-term exposure indicators, since the first reflects As exposure of 6 to 12 months prior to sample collection [58] and the second is produced from the 12th week of gestation [59]. Therefore, measurement of As in both samples could be a reliable source of exposure prior to GDM development.

The major limitation of this meta-analysis is the strong heterogeneity and degree of inconsistency existing between the nine individual risk estimates. Several differences between the included studies that may account for this heterogeneity were analyzed including study design, exposure indicator, study country, and diagnostic criteria of GDM. The random model estimator analysis did not substantially change the risk estimates and no reduction in heterogeneity among the included studies was observed when adopting this model. As a matter of fact, a fixed meta-analysis has natural complements that provide heterogeneity (i.e., Cochran’s Q), thus measures of heterogeneity should not be used to determine if this model could be appropriate [67,68]. Indeed, stratified analysis partially helped in understanding possible sources of heterogeneity. Nevertheless, the low number of studies included in the stratified analyses led to restricted statistical power and less precise risk estimates. Another limitation of this study is the significant publication bias indicated by the Funnel plot as a consequence of the exclusion of evidence from unpublished (i.e., grey literature) and non-English language studies. To assess the association between As exposure and risk of developing GDM, we deemed it more appropriate not to include studies providing poor replicable evidence. Additionally, it is known that scientific literature is predominantly biased toward positive results, of which many are unlikely to correspond to the reality and to be applicable worldwide [69]. These limitations suggest that the results should be interpreted with caution until validated by future research projects providing more detailed, well designed, and standardized data collection. 

## 5. Conclusions

In summary, the results of this systematic review and meta-analysis provide additional evidence for a possible association between As exposure and the risk of GDM. To improve and confirm the available data, future study designs might benefit from the inclusion of standardized methods with more sensitive limits of exposure detection in order to evaluate the effects of inorganic and organic As on glucose homeostasis during early pregnancy, hence prior to GDM diagnosis. Additionally, as controversy still surrounds the diagnosis of GDM, a universally endorsed diagnostic criteria could help in confirming the potential role of As in contributing to the onset of this disease, hopefully implying new prevention strategies to reduce the burden of GDM worldwide. 

## Figures and Tables

**Table 1:** Main characteristics of the considered studies.

Author, Year	Study Country	Study Design	Study Period	Sample Size (Cases/ Controls)	Age (Cases/ Controls)	Definition of Cases	Exposure Indicator and When	As Exposure (Cut-Off or LOD)	Confounding Factors Considered
• **Blood samples (3 studies)**									
Shapiro et al., 2015 [14]	Canada	Cohort study	2008–2011	48/1167	18–29 yo: 12.5%/ 24.8% 30–34 yo: 45.8%/ 34.8% ≥35 yo: 41.7%/ 40.2%	CDA-SOGC Criteria ^a^ [38,39]	1st trimester blood samples *^1^*	LOD: 0.22 µg/L	Maternal age, race, pre-pregnancy BMI, education, parity, race
Xia et al., 2018 [25]	China	Cohort study	05/2013– 09/2014	419/2841	cases: 27.79 ± 4.25 yo controls: 26.18 ± 3.48 yo	ADA Diagnostic Criteria ^b^ [35]	1st,2nd, 3rd trimester serum samples	LOD: 0.0047 µg/L	Maternal age, pre-pregnancy BMI, monthly income, gestational age, parity
Wang Y. et al., 2019 [26]	China	Cohort study	2012–2016	776/776	cases: 31.00 ± 4.53 yo controls: 30.97 ± 4.53 yo	ADA Diagnostic Criteria ^b^ [35]	Serum ^2^ samples the day before delivery	As level: a. Low <10.64 µg/L b. Middle 10.64–21.12 µg/L c. High ≥21.12 µg/L	Maternal age, pre-pregnancy BMI, gestational weight gain, physical activity, family history of diabetes, month of conception, residence, education, monthly income, smoking, fetal gender, parity, gestational age
• **Urine samples (5 studies)**									
Farzan et al., 2016 [27]	USA	Cohort study	01/2009– 05/2016	14/1032	cases: 32.2 yo controls: 30.9 yo	ADA Diagnostic Criteria ^b,c^ [35]	home tap water samples, urine samples at 24–28 gw, toenails samples	LOD (urine): 0.10–0.15 µg/L	Maternal age, pre-pregnancy BMI, pregnancy weight gain, smoking, secondhand smoke exposure, education, gestational week of glucose testing, urinary creatinine
Ashley-Martin et al., 2018 [28]	Canada	Cohort study	2008–2011	42/1049	<29 yo: 19.2%/ 30.3% 30–34 yo: 46.8%/ 36.3% ≥35 yo: 34.0%/ 33.7%	CDA-SOGC Diagnostic Criteria ^a^ [38,39]	1st trimester urinary concentrations of arsenite, arsenate, MMA, DMA and AsB	LOD: 0.75 µg/L	Maternal age, gravidity, race, education, parity, pre-pregnancy BMI, maternal first trimester blood Cd levels
Munoz et al., 2018 [29]	Chile	Cross-sectional study	06/2013– 10/2013	21/223	≤29 yo: 57.1%/ 74.4% 30–34 yo: 28.6%/ 15.7% ≥35 yo: 14.3%/ 9.9%	WHO Diagnostic Criteria ^d,e^ [36]	2nd trimester urinary levels of arsenite, arsenate, MMA, DMA, T-InAs (calculated by adding values of these species)	LOD: 0.1 µg/L	Maternal age, education, ethnicity, BMI
Khan et al., 2018 [30]	Bangladesh	Cross-sectional study	-	31/169	cases: 25.19 ± 4.28 yo controls: 23.95 ± 3.92 yo	WHO Diagnostic Criteria ^d^ [36]	urine samples (not said when)	Not As exposed: ≤0.100 mg/L As exposed: >0.100 mg/L	Maternal age, gestational age, parity, BMI
Wang X. et al., 2020 [31]	China	Cohort study	07/2014– 07/2016	241/1849	cases: 29.54 ± 4.13 yo controls: 28.25 ± 3.34 yo all sample: 28.40 ± 3.47 yo	ADA Diagnostic Criteria ^b^ [35]	urine samples *^3^* <20 gw	LOD: 0.009 µg/L CAU-As: a. Low <32.11 µg/L b. Middle 32.11–48.11 µg/L c. High ≥48.11 µg/L	Maternal age, pre-pregnancy BMI, gravidity, occupational status, smoking exposure, average personal monthly income, family history of diabetes, physical activity, fetal sex
• **Tap water samples (2 studies)**									
Farzan et al., 2016 [27]	USA	Cohort study	01/2009– 05/2016	14/1032	cases: 32.2 yo controls: 30.9 yo	ADA Diagnostic Criteria ^b,c^ [35]	home tap water samples, urine samples, toenails samples	LOD (water): 0.001–0.07 µg/L	Maternal age, pre-pregnancy BMI, pregnancy weight gain, smoking, secondhand smoke exposure, education, gestational week of glucose testing, urinary creatinine
Marie et al., 2018 [33]	France	Semi- ecological study (correlational)	2003 2006 2010	286/4767	all sample: 29.1 ± 5.6 yo	CNGOF Diagnostic criteria ^g^ [37]	water samples during the 12 months before pregnancy	Not As exposed: <10 µg/L As Exposed: a. Low 10–30 µg/L b. High ≥ 30µg/L	Maternal age, family situation, number of inhabitants in commune of residence, geographic origin, employment during pregnancy, paid employment, pre-pregnancy BMI, type of pregnancy, year of delivery
• **Meconium samples (1 study)**									
Peng et al., 2015 [32]	China	Case-control study nested in a cohort	06/2012– 07/2012	137/190	cases: 27.85 ± 3.87 yo controls: 26.34± 2.64 yo	WHO Diagnostic Criteria ^d,f^ [36]	meconium samples during the first 2 postnatal days	LOD: 0.06 µg/L	Maternal age, pre-pregnancy BMI, gravidity, parity, HBV infection, newborn sex
• **Toenails samples (1 study)**									
Farzan et al., 2016 [27]	USA	Cohort study	01/2009– 05/2016	14/1032	cases: 32.2 yo controls: 30.9 yo	ADA Diagnostic Criteria ^b,c^ [35]	home tap water samples, urine samples, toenails samples 2 weeks post-partum	Ln toenails As (µg/g) (not said LOD)	Maternal age, pre-pregnancy BMI, pregnancy weight gain, smoking, secondhand smoke exposure, education, gestational week of glucose testing, urinary creatinine

Abbreviations: As, Arsenic; LOD, limit of detection; GDM, Gestational diabetes mellitus; yo, years old; BMI, body max index; ADA, American Diabetes Association; CAU-As, creatinine-adjusted urinary arsenic; gw, gestational week; Cd, Cadmium; MMA, monomethylarsonic acid; DMA, dimethylarsinic acid; AsB, and arsenobetaine; T-InAs, Total inorganic arsenic; WHO, World Health Organization; CDA-SOGC, Canadian Diabetes Association-Society of Obstetricians and Gynecologist of Canada; CNGOF, French National College of Obstetricians and Gynecologists; HBV, Hepatitis B virus; Ln, Logarithm. Notes: ^1^ Arsenic, Cadmium, Mercury, Lead, Eleven phthalate metabolites and Total Bisphenol A. ^2^ Nickel, Arsenic, Cadmium, Antimony, Tallium, Mercury, and Lead. ^3^ Nickel, Arsenic, Antimony, Cadmium, Cobalt and Vanadium. ^a^ GCT 50 gr positive (After 1 h: >10.3 mmol/L) or OGTT 75/100 gr at least 2 altered values (Fasting: >5.3/5.8 mmol/L; After 1 h: >10.6 mmol/L; After 2 h: >8.9/9.2 mmol/L; After 3 h: -/8.0 mmol/L). ^b^ One step approach: OGTT 75 gr at 24–28 gw at least 1 altered value (Fasting: ≥5.1 mmol/L; After 1 h: ≥10.0 mmol/L; After 2 h: ≥8.5 mmol/L). ^c^ *Farzan* et al. (*2016*). Two step approach: GCT 50 gr at 24–28 gw high positive (After 1 h: >200 mg/dL) or GCT 50 gr at 24–28 gw borderline (After 1 h: 120–140 mg/dL)/positive (After 1 h: 140–200 mg/dL) and OGTT 100 gr at least 2 positive values (Fasting: ≥5.3 mmol/L; After 1 h: ≥10.0 mmol/L; After 2 h: ≥8.6 mmol/L; After 3 h: 7.8 mmol/L)/diagnosis of GDM in medical records. ^d^ GDM diagnosis: OGTT 75 gr at any time of pregnancy at least 1 altered value (Fasting: 5.1–6.9 mmol/L (92–125 mg/dL); After 1 h: 10.0 mmol/L (180 mg/dL); After 2 h: 8.5–11.0 mmol/L (153–199 mg/dL)).^e^ *Munoz* et al. (*2018*). Criteria established in Pregnancy and Diabetes Guide by the Ministry of Health of Chile according to WHO diagnostic criteria for diabetes: Blood glucose at early pregnancy on 2 different days positive (Fasting glycemia: 100–125 mg/dL) and/or OGTT 75 gr at 24–28 gw positive (After 2 h: ≥140 mg/dL).^f^ *Peng* et al. (*2015*). Diabetes in pregnancy diagnosis (more severe than GDM): OGTT 75 gr at any time of pregnancy at least 1 altered value (Fasting ≥7.0 mmol/L; After 2 h: ≥11.1 mmol/L).^g^ GCT 50 gr positive (After 1 h: ≥2.0 g/L) or GCT 50 gr borderline (1.30–2 g/L) and OGTT 100 gr at least 2 positive values (Fasting: >0.95 g/L; After 1 h: >1.80 g/L; After 2 h: >1.55 g/L; After 3 h: >1.40 g/L).

**Table 2:** Stratified meta-analysis of maternal as exposure and the risk of developing GDM.

Stratifications	*N*. Studies	Effect Estimates		Heterogeneity		
		OR	(95% CI)	χ^2^	*p*	*I^2^*
All included studies ^a,b^ [14,25,26,27,28,29,30,31,32,33]	9	1.56	(1.23, 1.99)	21.95	0.005	64%
All studies less Peng et al. (2015)	8	1.43	(1.17, 1.74)	14.28	0.05	51%
All studies less Farzan et al. (2016)	8	1.73	(1.27, 2.43)	19.46	0.007	64%
All studies less Wang Y. et al. (2019)	8	1.72	(1.30, 2.27)	18.55	0.01	62%
All studies less Ashley Martin et al. (2018)	8	1.50	(1.19, 1.89)	18.88	0.009	63%
All studies less Marie et al. (2018)	8	1.57	(1.20, 2.05)	21.32	0.003	67%
All studies less Munoz et al. (2018)	8	1.59	(1.24, 2.04)	21.85	0.003	68%
All studies less Shapiro et al. (2015)	8	1.47	(1.17, 1.84)	17.56	0.01	60%
All studies less Wang X. et al. (2020)	8	1.66	(1.23, 2.23)	21.89	0.003	68%
All studies less Xia et al. (2018)	8	1.55	(1.18, 2.04)	19.63	0.006	64%
Study design						
Cohort studies	5	1.16	(1.07, 1.26)	13.73	0.008	71%
Cross-sectional studies	2	2.28	(0.92, 5.64)	1.86	0.17	46%
Nested case-control studies	1	/	/	/	/	/
Correlational studies	1	/	/	/	/	/
Exposure indicator						
Blood samples	3	1.35	(1.11, 1.65)	8.87	0.01	77%
Urine samples	4	1.39	(1.07, 1.82)	4.20	0.24	29%
Tap water samples	2	1.11	(1.02, 1.21)	2.49	0.11	60%
Meconium samples	1	/	/	/	/	/
Toenails samples	1	/	/	/	/	/
Study country						
North America	3	1.28	(1.07, 1.53)	8.57	0.01	77%
North America less *Shapiro* et al. *(2015) ^c^*	2	1.23	(1.03, 1.48)	3.65	0.06	73%
Asia	4	1.37	(1.17, 1.62)	12.32	0.006	76%
Asia less *Peng* et al. *(2015) ^d^*	3	1.32	(1.12, 1.56)	4.78	0.09	58%
South America	1	/	/	/	/	/
Europe	1	/	/	/	/	/
Diagnostic criteria						
ADA	4	1.27	(1.12, 1.43)	5.37	0.15	44%
ADA less *Farzan* et al. *(2016) ^e^*	3	1.32	(1.12, 1.56)	4.78	0.09	58%
WHO	2	3.13	(1.41, 6.95)	3.36	0.07	70%
CDA-SOGC *^c^*	2	3.76	(1.79, 7.91)	0.00	0.96	0%
CNGOF	1	/	/	/	/	/

Abbreviations: As, Arsenic; GDM, Gestational Diabetes Mellitus; N. studies, Number of studies; OR, Odds Ratio; 95% CI, 95% Confidence Interval; ADA, American Diabetes Association; WHO, World Health Organization; CDA-SOGC, Canadian Diabetes Association-Society of Obstetricians and Gynecologist of Canada; CNGOF, French National College of Obstetricians and Gynecologists. Notes: ^a^ Forest plot in Figure 2. Funnel plot in Figure 3. ^b^ Sensitivity analyses were conducted by omitting one study at time. ^c^ Shapiro et al. (2015) and Ashley-Martin et al. (2018) extracted study participants from the Maternal-Infant Research on Environmental Chemicals (MIREC) longitudinal birth cohort, Canada. Because of possible redundancy between some data, stratified analysis according to study country (North America) was also performed by omitting the study Shapiro et al. (2015), whereas stratification according to diagnostic criteria of GDM (CDA-SOGC diagnostic criteria) needs to be interpreted with caution. ^d^ Peng et al. (2015) conducted a retrospective case-control study nested within a cohort using newborns’ meconium as exposure indicator. The study designs of Wang X. et al. (2020), Wang Y. et al. (2019), and Xia et al. (2018) were all prospective cohort studies based on maternal samples (respectively urine, blood, blood) as exposure assessment mode. In light of these methodological differences, analysis was also performed by omitting Peng et al. (2015). ^e^ Farzan et al. (2016) defined cases based on ADA diagnostic criteria according to the one step or the two step approaches. As all the other studies where diagnosis of GDM was made according to these criteria [25,26,31] considered only the one step approach, analysis was also performed by omitting Farzan et al. (2016).

## Supplementary Materials

The following are available online at https://www.mdpi.com/2072-6643/12/10/3094/s1, Figure S1: Risk of bias assessment, Figure S2: Forest and Funnel Plots of subgroups analyses.
