# Supplementary material for: Maternal Arsenic Exposure and Gestational Diabetes: A Systematic Review and Meta-Analysis
Source: Nutrients. 2020 Oct 11;12(10):3094. doi: 10.3390/nu12103094 (PMC7600218; doi:10.3390/nu12103094)

Supplementary Figure S1. Risk of bias assessment according to the risk of bias tool for cohort studies by Clarity group [40].

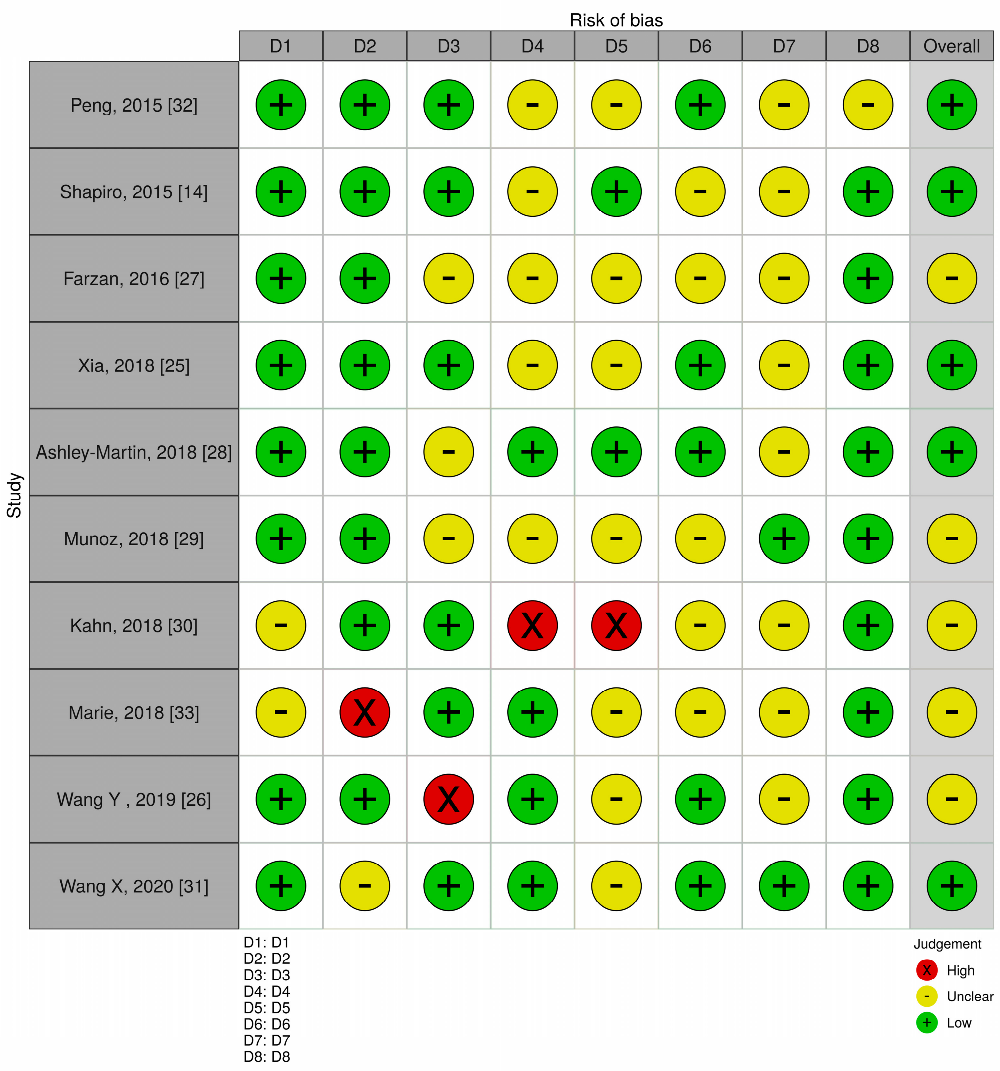

Supplementary Figure S2. Forest and Funnel Plots of subgroups analyses. (**a**) Study design: cohort; (**b**) Study design: cross-sectional; (**c**) Exposure indicator: Serum; (**d**) Exposure indicator: Urine; (**e**) Exposure indicator: Tap water; (**f**) Study country: North America; (**g**) Study country: Asia; (**h**) Diagnostic criteria: American Diabetes Association (ADA); (**i**) Diagnostic criteria: World Health Organization (WHO); (**j**) Diagnostic criteria: Canadian Diabetes Association-Society of Obstetricians and Gynaecologist of Canada (CDA-SOGC).

a) Study design: cohort

Study or Subgroup	log[Odds Ratio]	SE	Weight	Odds Ratio IV, Fixed, 95% CI
Farzan et al. (2016)	0.0953	0.0486	75.1%	1.10 [1.00, 1.21]
Shapiro et al. (2015)	1.3083	0.4959	0.7%	3.70 [1.40, 9.78]
Wang X. et al. (2020)	0.3293	0.148	8.1%	1.39 [1.04, 1.86]
Wang Y. et al. (2019)	0.077	0.1343	9.8%	1.08 [0.83, 1.41]
Xia et al. (2018)	0.5365	0.1687	6.2%	1.71 [1.23, 2.38]
**Total (95% CI)**			**100.0%**	**1.16 [1.07, 1.26]**

Heterogeneity: $Chi^2 = 13.73$, df = 4 (P = 0.008); $I^2 = 71\%$
Test for overall effect: Z = 3.53 (P = 0.0004)

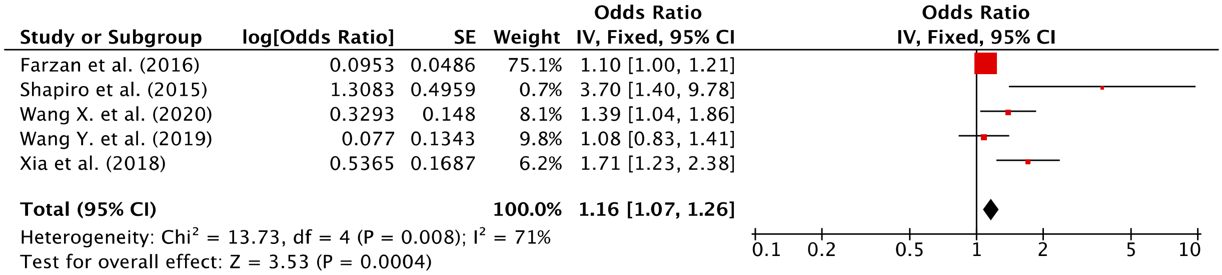

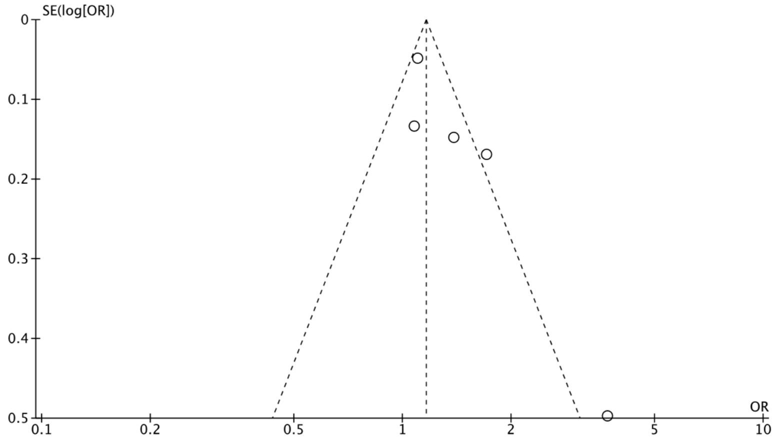

b) Study design: cross-sectional

Study or Subgroup	log[Odds Ratio]	SE	Weight	Odds Ratio IV, Fixed, 95% CI
Ashley Martin et al. (2018)	1.3507	0.6024	58.9%	3.86 [1.19, 12.57]
Munoz et al. (2018)	0.0677	0.7218	41.1%	1.07 [0.26, 4.40]
**Total (95% CI)**			**100.0%**	**2.28 [0.92, 5.64]**

Heterogeneity: $Chi^2 = 1.86$, df = 1 (P = 0.17); $I^2 = 46\%$
Test for overall effect: Z = 1.78 (P = 0.07)

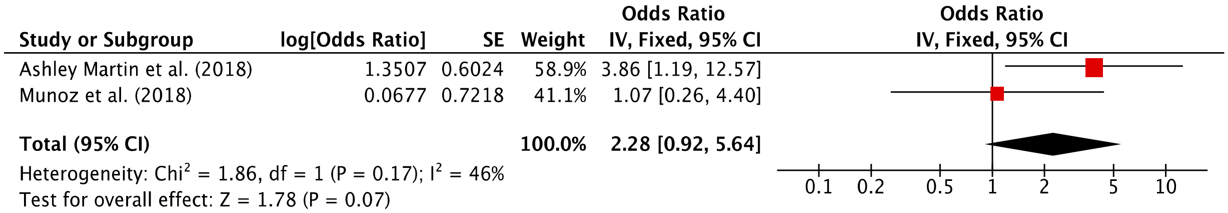

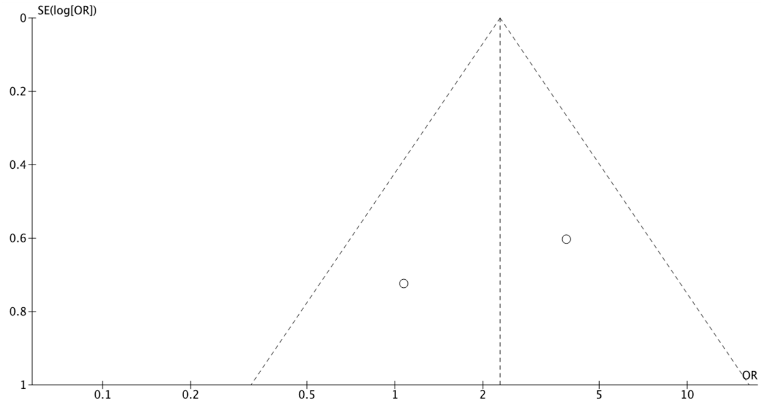

c) Exposure indicator: Serum

Study or Subgroup	log[Odds Ratio]	SE	Weight	Odds Ratio IV, Fixed, 95% CI
Shapiro et al. (2015)	1.3083	0.4959	4.3%	3.70 [1.40, 9.78]
Wang Y. et al. (2019)	0.077	0.1343	58.4%	1.08 [0.83, 1.41]
Xia et al. (2018)	0.5365	0.1681	37.3%	1.71 [1.23, 2.38]
**Total (95% CI)**			**100.0%**	**1.35 [1.11, 1.65]**

Heterogeneity: $Chi^2 = 8.87$, df = 2 (P = 0.01); $I^2 = 77\%$
Test for overall effect: Z = 2.93 (P = 0.003)

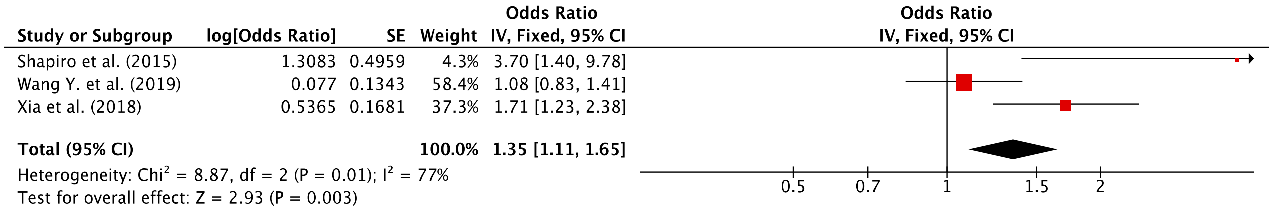

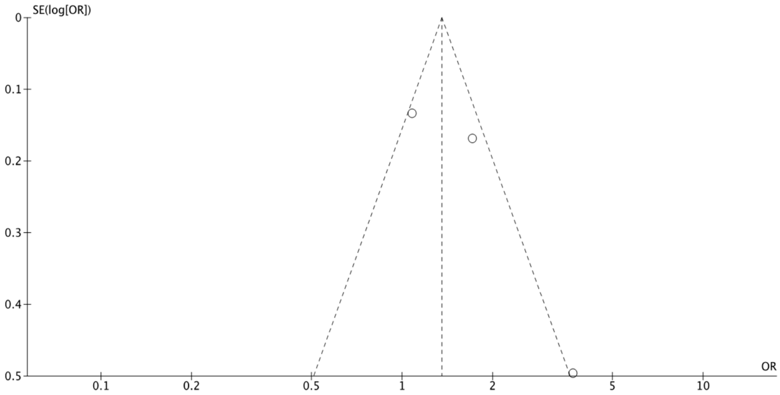

d) Exposure indicator: Urine

Study or Subgroup	log[Odds Ratio]	SE	Weight	Odds Ratio IV, Fixed, 95% CI
Ashley Martin et al. (2018)	1.3507	0.6047	5.0%	3.86 [1.18, 12.63]
Farzan et al. (2016)	−0.2231	0.5004	7.4%	0.80 [0.30, 2.13]
Munoz et al. (2018)	0.0677	0.7218	3.5%	1.07 [0.26, 4.40]
Wang X. et al. (2020)	0.3293	0.148	84.1%	1.39 [1.04, 1.86]
**Total (95% CI)**			**100.0%**	**1.39 [1.07, 1.82]**

Heterogeneity: $Chi^2 = 4.20$, df = 3 (P = 0.24); $I^2 = 29\%$
Test for overall effect: Z = 2.44 (P = 0.01)

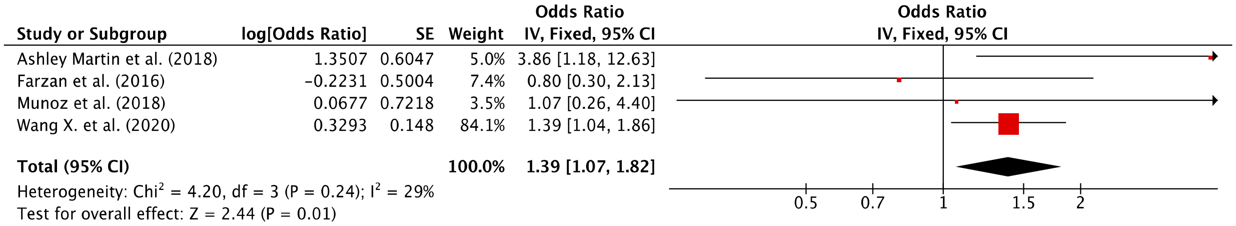

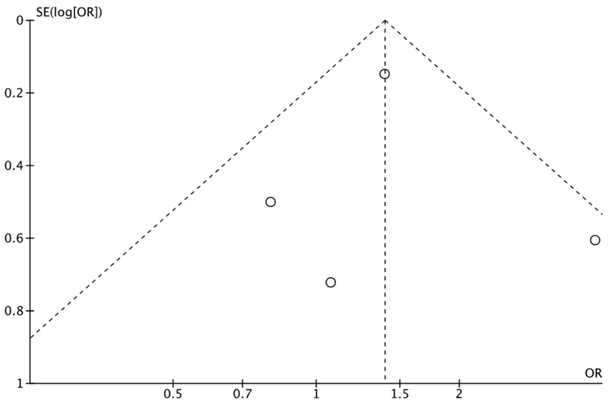

e) Exposure indicator: Tap water

Study or Subgroup	log[Odds Ratio]	SE	Weight	Odds Ratio IV, Fixed, 95% CI
Farzan et al. (2016)	0.0953	0.0444	96.7%	1.10 [1.01, 1.20]
Marie et al. (2018)	0.4824	0.2411	3.3%	1.62 [1.01, 2.60]
**Total (95% CI)**			**100.0%**	**1.11 [1.02, 1.21]**

Heterogeneity: Chi^2 = 2.49, df = 1 (P = 0.11); I^2 = 60%
Test for overall effect: Z = 2.47 (P = 0.01)

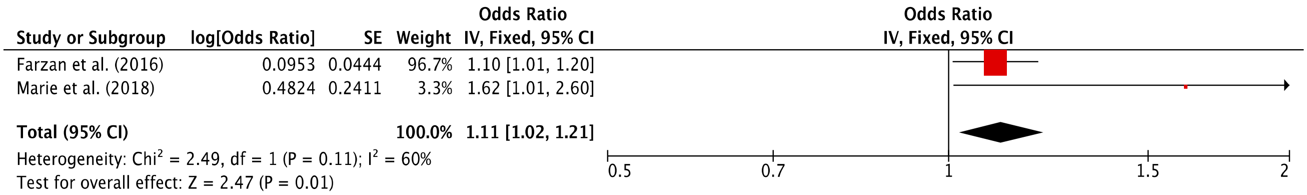

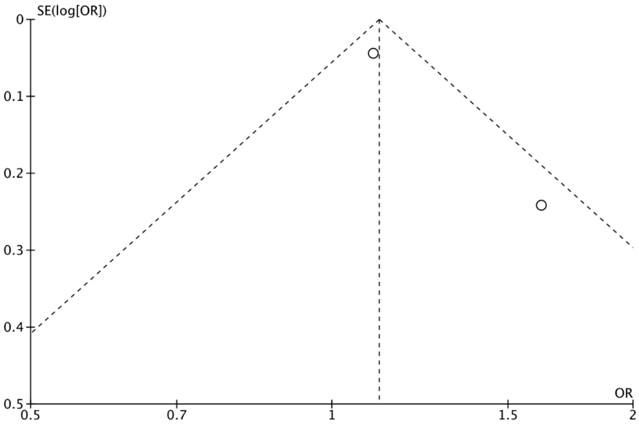

f) Study country: North America

Study or Subgroup	log[Odds Ratio]	SE	Weight	Odds Ratio IV, Fixed, 95% CI
Ashley Martin et al. (2018)	1.3507	0.6047	2.2%	3.86 [1.18, 12.63]
Farzan et al. (2016)	0.1823	0.093	94.3%	1.20 [1.00, 1.44]
Shapiro et al. (2015)	1.3083	0.4865	3.4%	3.70 [1.43, 9.60]
**Total (95% CI)**			**100.0%**	**1.28 [1.07, 1.53]**

Heterogeneity: $Chi^2 = 8.57$, df = 2 (P = 0.01); $I^2 = 77\%$
Test for overall effect: Z = 2.74 (P = 0.006)

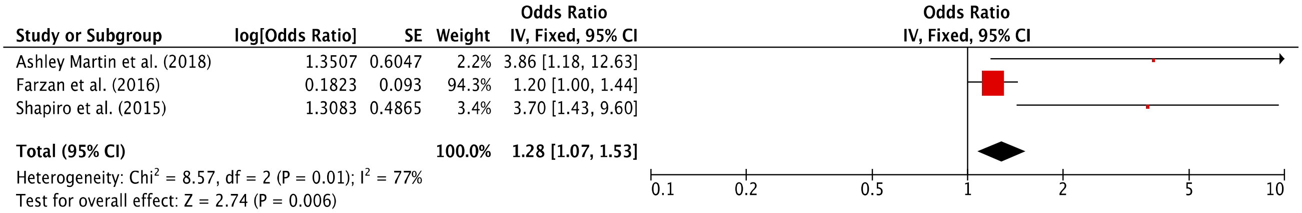

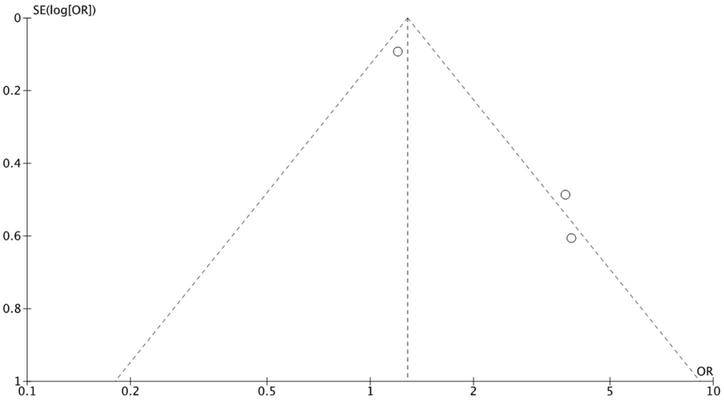

g) Study country: Asia

Study or Subgroup	log[Odds Ratio]	SE	Weight	Odds Ratio IV, Fixed, 95% CI
Peng et al. (2015)	1.6582	0.4953	2.8%	5.25 [1.99, 13.86]
Wang X. et al. (2020)	0.3293	0.1431	33.7%	1.39 [1.05, 1.84]
Wang Y. et al. (2019)	0.077	0.1324	39.3%	1.08 [0.83, 1.40]
Xia et al. (2018)	0.5365	0.1687	24.2%	1.71 [1.23, 2.38]
**Total (95% CI)**			**100.0%**	**1.37 [1.17, 1.62]**

Heterogeneity: $Chi^2 = 12.32$, df = 3 (P = 0.006); $I^2 = 76\%$
Test for overall effect: Z = 3.83 (P = 0.0001)

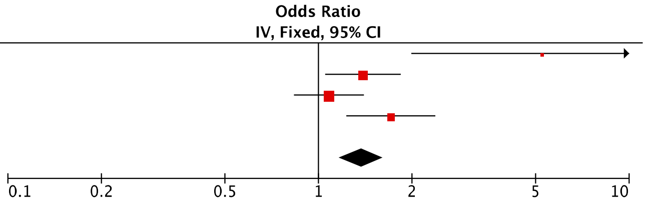

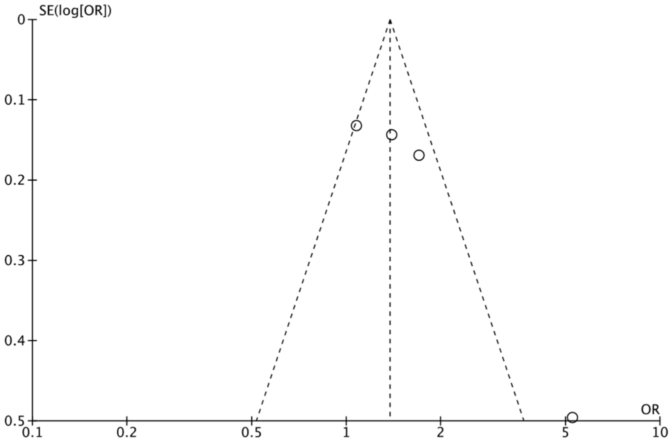

h) Diagnostic criteria: American Diabetes Association (ADA)

Study or Subgroup	log[Odds Ratio]	SE	Weight	Odds Ratio IV, Fixed, 95% CI
Farzan et al. (2016)	0.1823	0.093	45.1%	1.20 [1.00, 1.44]
Wang X. et al. (2020)	0.3293	0.1431	19.0%	1.39 [1.05, 1.84]
Wang Y. et al. (2019)	0.077	0.1324	22.2%	1.08 [0.83, 1.40]
Xia et al. (2018)	0.5365	0.1687	13.7%	1.71 [1.23, 2.38]
**Total (95% CI)**			**100.0%**	**1.27 [1.12, 1.43]**

Heterogeneity: $Chi^2 = 5.37$, df = 3 (P = 0.15); $I^2 = 44\%$
Test for overall effect: Z = 3.77 (P = 0.0002)

Odds Ratio
IV, Fixed, 95% CI

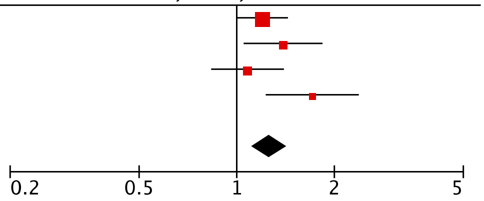

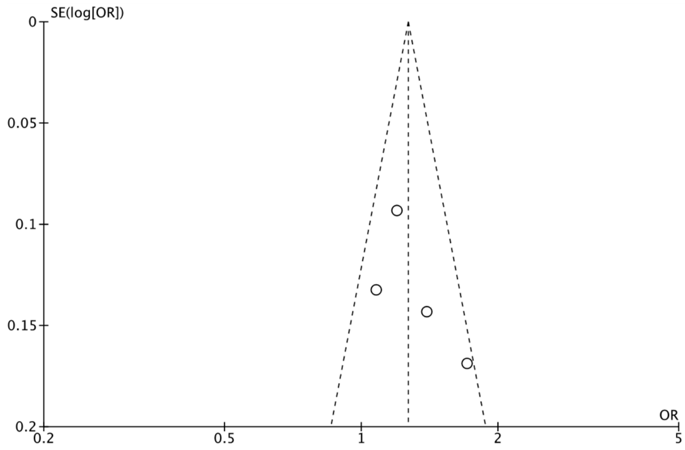

i) Diagnostic criteria: World Health Organization (WHO)

Study or Subgroup	log[Odds Ratio]	SE	Weight	Odds Ratio IV, Fixed, 95% CI
Munoz et al. (2018)	0.0677	0.7132	32.5%	1.07 [0.26, 4.33]
Peng et al. (2015)	1.6582	0.4953	67.5%	5.25 [1.99, 13.86]
**Total (95% CI)**			**100.0%**	**3.13 [1.41, 6.95]**

Heterogeneity: $Chi^2 = 3.36$, df = 1 (P = 0.07); $I^2 = 70\%$
Test for overall effect: Z = 2.80 (P = 0.005)

Odds Ratio
IV, Fixed, 95% CI

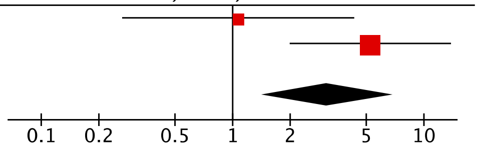

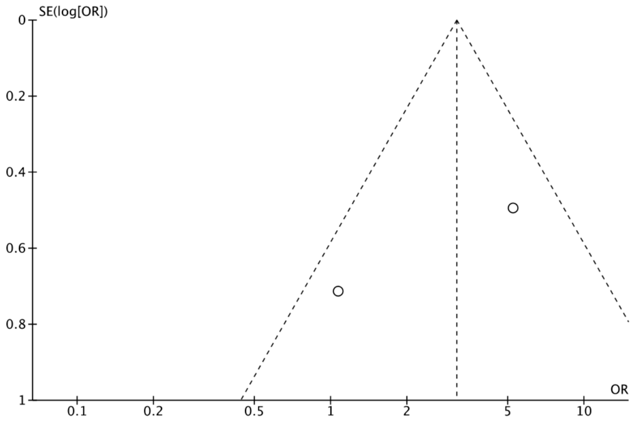

j) Diagnostic criteria: Canadian Diabetes Association-Society of Obstetricians and Gynaecologist of Canada (CDA-SOGC)

Study or Subgroup	log[Odds Ratio]	SE	Weight	Odds Ratio IV, Fixed, 95% CI
Ashley Martin et al. (2018)	1.3507	0.6047	39.3%	3.86 [1.18, 12.63]
Shapiro et al. (2015)	1.3083	0.4865	60.7%	3.70 [1.43, 9.60]
**Total (95% CI)**			**100.0%**	**3.76 [1.79, 7.91]**

Heterogeneity: $Chi^2 = 0.00$, df = 1 (P = 0.96); $I^2 = 0\%$
Test for overall effect: Z = 3.50 (P = 0.0005)

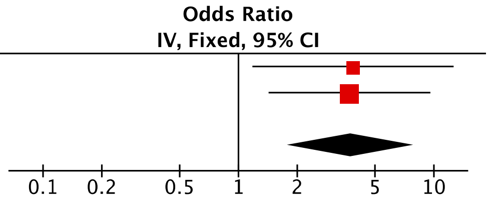

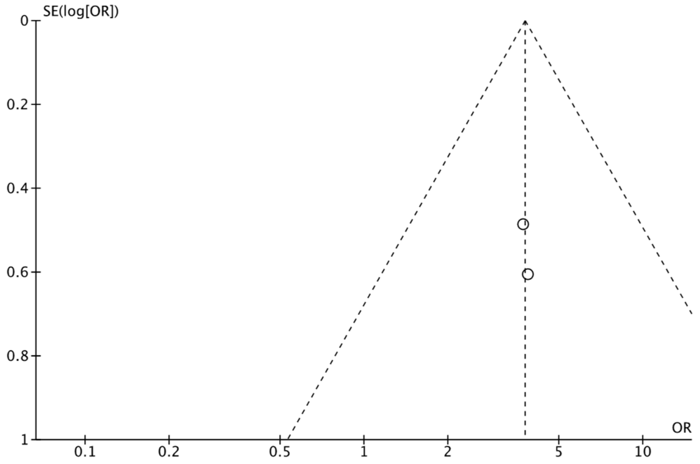

Supplement: Supplementary file 1 [file nutrients-12-03094-s001.pdf]
